# Neglected epidemics: The role of oral public health to advance global health

**DOI:** 10.7189/jogh.12.02001

**Published:** 2022-05-21

**Authors:** Heikki Murtomaa, Benoit Varenne, Prathip Phantumvanit, Usuf Chikte, Mohammad Hossein Khoshnevisan, Nadereh Mousavi Fatemi, Hossein Hessari, Mohammad Reza Khami

**Affiliations:** 1 Department of Oral and Maxillo-facial Diseases, University of Helsinki, Helsinki, Finland; 2 Department of Non-Communicable diseases, World Health Organization, Geneva, Switzerland; 3 School of Dentistry, Thammasat University, Bangkok, Thailand; 4 Department of Global Health, University of Stellenbosch, Stellenbosch, South Africa; 5 WHO-Collaborating Center for Training and Research in Dental Public Health, Tehran, Iran; 6 Oral Health Bureau, Ministry of Health and Medical Education, Tehran, Iran; 7 Research Center for Caries Prevention, Dentistry Research Institute, and Department of Community Oral Health, School of Dentistry, Tehran University of Medical Sciences, Tehran, Iran

This was the first time that an oral health panel was included in the agenda of World Health Summit meetings. The present document is the report of the panel of “Neglected Epidemics: The Role of Oral Public Health to Advance Global Health” in the 7th Regional World Health Summit (WHS), 29-30 April, 2019 Kish Island, Iran. This seems to be an important crucial step for further improvement of oral health as a significant requirement for overall health promotion at the regional and global level. The panel members prepared and published a declaration which has been published in the WHS June 2020 newsletter. We write to you in order to convey the messages of the panel to a wider audience community.

Oral diseases represent a major public health problem due to their high prevalence regionally and globally. According to a recent study on global burden of diseases, untreated dental caries was the most prevalent condition among the 313 diseases assessed globally [1-3]. Oral diseases impose heavy financial burden not only in low income countries, but also in high- and middle-income countries. According to World Health Organization, oral diseases are the fourth most expensive condition to treat, often surpassing the expenses for cancer, heart disease, stroke and dementia treatments, and causing negative impact on individual’s quality of life [4]. Fortunately, ample sound scientific evidence is available on effective preventive methods and promoting programs to prevent major oral diseases and maintaining good oral health at the individual and community levels.

Given that most oral diseases like other non-communicable diseases (NCDs) are behavioural related conditions, individuals are expected to play an active role for their own health improvement. To support a good oral health behaviour, in addition to providing sufficient oral self-care knowledge, proper access to enabling environment as well as preventive health system should be made available where people grow up, live and work. Unfortunately, in many countries the opportunities for community-based activities in oral health promotion are either limited or underutilized.

Recent scientific findings have brought better understanding on the important role of good oral health in overall health and even treatment of major NCDs. Chronic oral infection has close relation with diseases like atherosclerosis, heart diseases, respiratory diseases, malignancies and pregnancy complications such as premature births and low birth-weight [5-9]. It has also been well documented that diabetes and chronic kidney disease (CKD) have two-way relationship with untreated gum disease [10-12]. Without prevention and proper treatment (eradication of oral infections) both systemic conditions will further intensify the burden of oral diseases.

**Figure:**
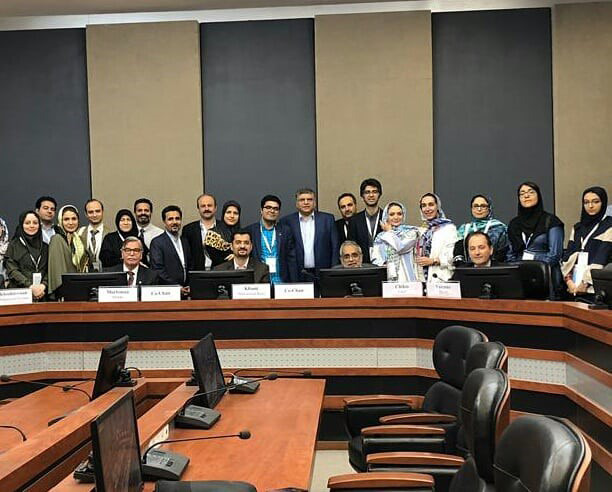
Photo: Oral health panel, at World Health Summit.

On the other hand, oral diseases share the same risk factors with major NCDs as described in Common Risk Factor Approach. Conditions such as cardiovascular diseases, chronic respiratory diseases, cancers and diabetes as well as obesity are like oral diseases: all affected by tobacco use, harmful use of alcohol, and unhealthy diet which are mostly preventable [13-17]. Successful prevention of oral diseases can reduce the burden of NCDs at the national, regional and global levels. Inter-professional collaboration can perfectly pave the way for controlling NCD’s common risk factors and improving not only the oral health but promoting the overall health and well-being.

Oral health professionals including dentists, dental therapists, hygienists, and dental academics, together with other health professionals can play more effective role in controlling the burden of NCDs at the community level, as emphasized in UN Sustainable Development Goals (SDG17) [18]. Delegating prevention and essential primary oral health care to oral health auxiliaries and in some extend to community (or primary) health workers can successfully control oral diseases in dental facilities, schools and communities to improve and promote oral health effectively.

The presentations and discussions in oral health panel at the World Health Summit, Regional Meeting of 2019, deemed the followings items important enough for improving and maintaining good oral health. Therefore, requesting kind attention of all health care providers and policy makers to the following points:

1. Oral diseases as a neglected NCD is currently considered a “silent epidemic”, putting heavy burden on general health, deserving special attention by all relevant constituents.
2. Oral health is a fundamental human right and it should be an integral part of Universal Health Coverage (UHC) within the global health agenda.
3. Oral health strategies should be included in all health policies.
4. To address essential oral health needs of a population through UHC, adequately trained oral health personnel with appropriate educational backgrounds should be available.
5. In order to address Sustainable Development Goals (SDGs), a proper oral health (SDG 3) [18] for all social strata (SDG 10) [17], through well-organized oral health services including primary health care (PHC) is essential.
6. In line with Common Risk Factor Approach, preventing the consumption of high sugar intake of foods and drinks, tobacco use, and harmful use of alcohol are particularly recommended.
7. The health care professionals should encourage good oral health knowledge and practice for prevention of oral diseases. Therefore, oral health should be included in the training of all medical professional.
8. Considering the high prevalence of oral diseases in the Eastern-Mediterranean Region, the NCD departments should include and support oral health strategies with high priority.
9. The 21 countries in the Eastern-Mediterranean Region would highly benefit, if a regional oral health advisor is appointed at the regional office (EMRO).
10. Innovative oral public health research should be supported equally if not more than clinical research, with more priority and sufficient resources by the Ministries of Health, dental schools and research institutions.
